# Home care utilization and outcomes among Asian and other Canadian patients with heart failure

**DOI:** 10.1186/1471-2261-10-12

**Published:** 2010-03-04

**Authors:** Guanmin Chen, Nadia Khan, Kathryn M King, Brenda R Hemmelgarn, Hude Quan

**Affiliations:** 1 Department of Community Health Sciences, University of Calgary, Canada; 2 The Center for Health and Policy Studies, University of Calgary, Canada; 3 The Center for Health Evaluation and Outcome Sciences, University of British Columbia, Canada; 4 Department of Medicine, University of Calgary, Canada

## Abstract

**Background:**

Heart failure (HF) is a major cause of hospitalization and death in the aging population around the world. Home care utilization is associated with improved survival for the patients with HF, and varies by ethno-culture. The purpose of this study was to investigate the difference in hospital readmission rate and mortality between Asian and other Canadian HF patients.

**Methods:**

HF patients were identified using hospital discharge abstracts from March 31, 2000 to April 1, 2006 in Calgary Health Region. Readmission and one-year mortality for HF were determined by linking hospital discharge and vital statistics data. Stratified by home care services use, readmission and mortality rates were compared between the Asians and other Canadians while controlling for age, sex, comorbidities, and household income.

**Results:**

Of 26,171 HF patients discharged from hospital, 56.6% of Asians and 58.0% of other Canadians used home care services [adjusted odds ratio (OR) for Asian: 0.84, 95% confidence interval (CI): 0.74-0.89]. The hospital readmission rate was similar between Asians and other Canadians regardless of home care services use. Mortality was similar between those who used home care services (adjusted OR for Asian: 0.96, 95% CI: 0.81-1.13). For patients who did not use home care services, Asians had significantly lower mortality than other Canadians (adjusted OR for Asian: 0.76, 95% CI: 0.60-0.86).

**Conclusion:**

Mortality was similar between Asian and other Canadian patients when home care services were utilized. However, among those without home care, Asian patients had a significantly lower mortality than other Canadian patients.

## Background

Heart failure (HF) is a major cause of hospitalization and death in the aging population around the world. Approximately 6-10% of the elderly population[1] has HF which accounts for more than 5% of medicine and geriatric admissions to hospital[2]. About 90% of HF patients discharged from hospital need home care service from either formal care providers; informal providers including family members;or friends or both[3]. Because of the aging population and greater survival of HF patients[4,5], more people with HF and its complications will require more home care and community-based services; potentially generating additional burdens for family members trying to provide the substantial daily care required by these individuals[6].

The racial and ethnic disparities in health services utilization and outcomes have been extensively studied[7]. Findings from previous research indicate that Black, Hispanic, and some Asian American populations have higher rates of chronic disease[8], are less likely to utilize health care, and experience more barriers of access to healthcare system than Whites[9]. Although Black, Hispanic and Asian Americans have lower nursing home services utilization[10,11], the ethnic users consume home care resources more intensively than White users[12,13]. A United States based study of patients in a nursing home demonstrates that elderly Asian Americans patients rely more heavily on family members to provide medical decision-making than other populations[10].

Previous Canadian studies have found that Asians have better outcomes than other Canadians for several chronic diseases. For example, Asians have a lower likelihood of death for end-stage renal disease [14] than other Canadians. Other recent studies indicate that home care services are associated with improved survival for the patients with HF [15,16]. In this study, we investigated the home care utilization and outcomes of Asians relative to other Canadian HF patients, using health administrative data.

## Methods

### Data source

Hospital discharge abstract, home care service, and vital statistics and Alberta Health Care Insurance Plan (AHCIP) databases in Calgary Health Region, Canada were employed in this study. Because of the Canadian universal insurance system, these databases included nearly all of 1.4 million residents in the region and are linked through a unique identifier, the Personal Health Number (PHN). The features of these databases include:

1. The hospital discharge abstract database contains demographic and clinical information for all patients discharged from hospital in the region. Prior to 2001, each discharge record was coded using International Disease Classification, 9^th ^Revision Clinical Modification (ICD-9-CM). Since 2001, each record was coded using ICD-10 Canadian Modification (ICD-10-CA).

2. The home care service data have been collected through the Home Care Reporting System (HCRS). We used this database to determine the patients using or not using formal home care services within 365 days after the index hospital discharge. Thus "home care services use" in this study was identified as formal home care service, provided by Alberta Health Service and captured through HCRS. The informal home care service provided by the family members were not measured or examined in this study.

3. The vital statistics database includes information on demographic characteristics, date of death, and causes of death. ICD-9-CM was employed to record cause of death for fiscal year 2000, and ICD-10-CA was used thereafter.

4. The Alberta Health Care Insurance Plan (AHCIP) registry contains registrants' name, date of birth, sex, and mailing address. The registry is frequently updated and considered a proxy for census data. The population in AHCIP was very similar to that in the Alberta census population[17].

We used data generated between April 1, 2000 and March 31, 2006 for our analyses.

### Study Population

Heart failure patients were defined using the hospital discharge abstract data. We extracted all admissions with validated primary diagnosis codes of 4254, 4255, 4257, 4258, 4258, and 428 for ICD 9CM and I43, I50, I099, I110, I132, I255, I420, I425-I429, and P290 for ICD 10 CA[18]. We assigned the first discharge date to each patient as index date. Patients younger than 20 years old, non-residents of Alberta, and those residing in nursing homes were excluded.

### Study Variables

We linked study population with AHCIP data and determined their ethnicity using surname methodology. Chinese was defined using the surname algorithm developed by Quan, et al.[19] and Japanese, Korean, Vietnam, Filipino, or South Asian Indian were defined using the surname algorithm developed by Lauderdale, et al.[20]. These ethnicities were grouped as "Asian" in this study. The remaining patients were classified as other Canadian.

Home care services utilization within one year after discharge was determined by linking the PHN with the HCRS database. Each patient was identified as either using or not using home care services.

The outcomes of interest in this study were hospital readmission and mortality within 365 days after index discharge date. Readmission was defined through linking study population with hospital discharge abstract data. Mortality after discharge was determined by linking the study population with the vital statistics registry using a deterministic record linkage method[21].

Potential confounding factors were identified. These included sex, age, annual household income, and comorbidities. Sex and age recorded in the hospital discharge data were used in the analysis. The annual household income of patients was derived from the Canadian Census 2001 median income file after patient's postal codes were converted into Census Enumeration area using the Statistics Canada Postal Code Conversion file [22]. This income reflects the area-level income, not precisely measuring an individual's actual income.

However, it is a valid and widely used method to impute individual socioeconomic status[23,24]. The presence of comorbidities (i.e., those identified in the Charlson Cormorbidity Index) were defined by the enhanced ICD-9-CM and ICD-10 coding algorithms [25]. The Charlson Index Score was calculated for each patient[26], and was used to adjust for comorbidities between the patients in this study.

### Statistical Analysis

Chi-square analyses were employed to test the difference of patient's characteristics based on ethnicity. The crude and risk adjusted odds ratio (OR) and their 95% confidential intervals (CI) for Asian relative to other Canadian HF patients for home care service utilization, readmission and mortality in the year following the index discharge, were estimated using logistic regression models. The data analyses were performed using SAS statistical software (version 9.13).

The study was reviewed and approved by the ethics board of the University of Calgary.

## Results

### Characteristics of sample

Of 26,171 HF patients, 1,182 (4.5%) were identified as Asian and 24,989 (95.5%) were identified as other Canadian. The distributions were significantly different between the Asian and other Canadian HF patients for sex (P < 0.001), age (P < 0.001), Charlson Index Score (P = 0.04), and annual household income (P < 0.001) (Table 1).

**Table 1:** Characteristics of Heart Failure Patients

	Asian		Other Canadian		P value
			
	N	Percent	N	Percent	
**Total**	1,182	100.0	24,989	100.0	~
**Sex**					
Male	547	46.3	13,485	54.0	<0.001
Female	635	53.7	11,504	46.0	
**Age (year)**					
≤ 65	244	20.6	6,587	26.4	<0.001
65-	306	25.9	5,299	21.2	
75-	421	35.6	7,991	32.0	
≥ 85	211	17.9	5,112	20.5	
**Charlson Index Score**					
0	153	12.9	3,472	13.9	0.04
1	277	23.4	6,136	24.6	
2	267	22.6	6,104	24.4	
3	188	15.9	3,852	15.4	
≥ 4	297	25.1	5,425	21.7	
**Income($, thousand)^#^**					
<30	190	16.1	4,661	18.7	<0.001
30-	240	20.3	7,502	30.0	
50-	522	44.2	9,340	37.4	
≥ 80	230	19.5	3,486	14.0	

#: Annual neighborhood median income

After adjustment for sex, age, annual household income and Charlson Index Score, Asian patients were less likely to utilize home care service than other Canadian patients (adjusted OR for Asian patients: 0.84, 95% CI: 0.74-0.89) (Table 2). In females, those patients who were more than 75 years old, or those patients who had an annual household income of less than 50 thousand dollars, Asians had a significantly lower home care utilization rate than other Canadian(P < 0.05). There were no significant differences for home care utilization between Asian and other Canadian male patients, those who were less than 75 years old, or those who had an annual household income more than 50 thousand dollars. The Asian and other Canadian HF patients had similar home care utilization when stratified for Charlson Index Score.

**Table 2:** Home Care Services Use in Asian and other Canadians

Groups	Home care service (%)		OR(95% CI, Asian vs. other Canadian)	
		
	Asian	Other Canadian	Crude	Adjusted
**Total**	56.6	58.0	0.94(0.84-1.06)	0.84(0.74-0.89)
**Sex**				
Male	44.8	48.8	0.85(0.82-1.01)	0.88(0.73-1.07)
Female	66.0	70.4	0.82(0.69-0.96)	0.80(0.67-0.97)
**Age (year)**				
≤ 65	24.7	34.8	0.61(0.47-0.81)	0.93(0.69-1.23)
65-	51.2	56.9	0.79(0.63-1.02)	0.83(0.65-1.06)
75-	62.0	69.2	0.73(0.59-0.89)	0.62(0.50-0.77)
≥ 85	78.7	83.7	0.72(0.51-1.01)	0.70(0.49-0.99)
**Charlson Index Score**				
0	45.8	53.1	0.75(0.54-1.03)	1.00(0.69-1.45)
1	50.9	52.3	0.95(0.75-1.21)	0.93(0.70-1.22)
2	54.7	55.0	0.99(0.77-1.26)	0.96(0.88-1.23)
3	59.0	67.6	0.89(0.51-1.09)	0.98(0.73-1.44)
≥ 4	63.9	68.0	0.83(0.65-1.08)	0.87(0.67-1.13)
**Income($, thousand)^#^**				
<30	57.1	73.3	0.49(0.34-0.68)	0.47(0.33-0.69)
30-	51.7	61.7	0.67(0.51-0.86)	0.72(0.54-0.95)
50-	59.0	60.2	0.95(0.80-1.14)	0.95(0.78-1.15)
≥ 80	60.9	59.8	1.05(0.80-1.38)	1.04(0.76-1.42)

#: Annual neighborhood median income

The readmission to hospital rates were similar across ethnicities and home care services utilization status (Table 3). Compared with other Canadians, the adjusted OR of readmission hospitalization for Asian patients was 0.98 (95% CI: 0.87-1.20) among those who used home care services and 0.94 (95% CI: 0.83-1.26) among patients who did not use home care services.

**Table 3:** Readmission to hospital and mortality rates for Asians compared to other Canadians

	Rate (%)		OR(95% CI, Asian vs. other Canadian)	
		
	Asian	Other Canadian	Crude	Adjusted
**Readmission**				
With home care service	49.7	50.3	0.97(0.88-1.20)	0.98(0.87-1.20)
Without home care service	26.7	29.2	0.93(0.93-1.38)	0.94(0.83-1.26)
**Mortality**				
With home care service	6.7	7.1	0.92(0.75-1.58)	0.96(0.81-1.13)
Without home care service	5.8	6.3	0.91(0.80-0.98)	0.76(0.60-0.86)

# adjusted for age, sex, household income and Charlson Index Score

There was no statistically significant difference in mortality between the Asian and other Canadian HF patients who utilized home care services (6.7% Asian versus 7.1% other Canadian, adjusted OR: 0.96, 95% CI: 0.81-1.13). For the patients who do not use home care services, the crude mortality was significantly different between the two groups (5.8% Asian versus 6.3% other Canadian, crude OR: 0.91, 95% CI: 0.80-0.98). After adjusting for sex, age, annual household income and Charlson Index Score, Asian patients still had significantly lower mortality than other Canadians among those who did not use home care services (adjusted OR: 0.76 for Asian patients, 95% CI: 0.60-0.86).

## Discussion

We, in this population-based study, investigated whether the outcomes of HF differed between Asian and other Canadian patients based on home care service utilization status. We found among the patients who did not receive home care services, that Asians had significantly lower mortality than other Canadians. But both ethnic groups were similar in mortality when they received home care service, and had similar hospital readmission regardless of home care services utilization status.

Previous study has demonstrated lower long term mortality among Asian patients with end-stage renal disease compared with patients of other Canadian[14]. In our study, there were no significant differences in mortality between Asian and other Canadian HF patients who received formal home care services. However, there was a significant difference in mortality between the ethnic groups who did not receive formal home care. This finding remained constant even after adjusting for age, sex, and annual household income, and Charlson Index Score. Although differences in severity of HF or utilization of secondary prevention therapies between the ethnic groups who did not utilize home care services may contribute to the differences in the observed mortality, another potential explanation is that Asian patients might receive more informal care (i.e., from family) than other Canadian patients.

Family support may facilitate better adherence to medical therapy, increased ability to attend outpatient physician appointments, and may be associated with less depression and isolation[3]. The Asian culture (including both Chinese and South Asians), places high value upon the extended family. Further, a significant part of Asian culture is filial piety, where family members are obliged to care for one another. In contrast, a significant part of European culture (found predominantly in other Canadians) is filial affection, whereby family members care for one another from affection rather than obligation[27,28]. Previous studies have indicated that elderly Asian people receive more informal home care than those who are White [10,29,30], and the informal home care from a family member is associated with improvement in HF outcomes after hospital discharge[29,31]. Another possible explanation is that Asian patients might adhere to medication regimens or comply with physician recommendations better than other Canadian patients.

Rates of readmission to hospital were similar between Asian and other Canadian patients regardless home care services utilization. Previous studies demonstrated that home care services are associated with reduced hospital readmission[32,33], while another study indicated that home care services do not have association with hospital readmission for elderly patients with HF[3]. The aim of the Canadian universal healthcare system is to provide equal access to health care system. Analysis of Canadian national survey demonstrated that the rate of physician and hospital utilization is stable across races/ethnicities[7]. Our finding of similar readmission between the two groups provides further evidence that racial/ethnic disparity in hospitalization is minor in Canada.

The similar mortality between the ethnic groups in patients who had home care services indicates home care services provided by professionals might indeed offer optimal benefit. An alternative explanation is that those who received formal home care services receive a similar level informal home care. Therefore, the mortality was not significantly different between Asian and other Canadian patients with home care service in our study.

There are several limitations in this study. First, the informal home care was not measured. We only investigated the association of formal home care service use and HF outcomes in this study. The association of informal home care (and the nature of that care) and HF outcomes between Asians and other Canadians still needs further study. Second, we did not analyze sub-ethnic groups under Asians and other Canadians. After excluded Spanish and aboriginals, the populations were almost White, and African-Americans were tiny among group of other Canadian. The largest Asian ethnic populations were Chinese and South Asians in our study and these ethnic sub-groups may have differences in the outcomes under study. Due to the small sample size for these ethnic sub-groups, we combined Chinese and South Asians as one. However, both South Asian and Chinese cultures strongly ascribe to filial piety and value in family support. Third, ethnicity was defined using surname lists and some patients' ethnicity may be misclassified. In addition, the marriage between Asians and other Canadians who adopt family names may also lead to misclassification of Canadians as Asian, and Asians as Canadians. In a previous survey, we found that a small proportion of Chinese women had an inter-racial marriage. Among of them, a majority did not change their surname after marriage [20]. However, we do not know if this is so for other Asian women. This potential misclassification could potentially bias the differences between the ethnic groups in this study.

## Conclusions

In conclusion, Asian patients with heart failure less often utilized home care services compared with patients of other Canadian. Asian patients had a lower mortality after discharge among those who did not receive home care services. Our findings suggest that other factors including utilization of informal home care may be contributing to the lower mortality beyond use of home care services. However, further study is needed to explore the role of informal home care in mortality among aging population.

## List of abbreviations

AHCIP: Alberta Health Care Insurance Plan; CI: confidence interval; HCRS: Home Care Reporting System; HF: heart failure; ICD: International Disease Classification; OR: odds ratio; PHN: person health number;

## Competing interests

The authors declare that they have no competing interests.

## Authors' contributions

Conception and design (HQ, GC), Analysis and interpretation of data (GC, HQ, NK, KMK, and BH), drafting the article (GC, HQ). All authors revised, read, and approved the final manuscript to be published.

## Pre-publication history

The pre-publication history for this paper can be accessed here:

http://www.biomedcentral.com/1471-2261/10/12/prepub
